# Acupuncture as adjunctive therapy for acute renal colic caused by urinary calculi: study protocol for a randomized controlled trial

**DOI:** 10.1186/s13063-021-05600-2

**Published:** 2021-09-25

**Authors:** Ying Cao, Jian-Feng Tu, Guang-Xia Shi, Li-Qiong Wang, Lian-Cheng Jia, Bo Li, Bao-Li Liu, Wei-Hai Yao, Xiao-Lu Pei, Zhi-Cheng Qu, Cun-Zhi Liu

**Affiliations:** 1 Emergency Department, Beijing Hospital of Traditional Chinese Medicine, Capital Medical University, 23 Meishuguanhou Street, Dongcheng District, Beijing, 100010 China; 2 International Acupuncture and Moxibustion Innovation Institute, School of Acupuncture-Moxibustion and Tuina, Beijing University of Chinese Medicine, No. 11, Bei San Huan Dong Lu, Chaoyang District, Beijing, 100029 China; 3 Urinary Surgery, Beijing Hospital of Traditional Chinese Medicine, Capital Medical University, Dongcheng District, Beijing, China; 4 Evidence Based Medicine Center, Beijing Hospital of Traditional Chinese Medicine, Capital Medical University, Dongcheng District, Beijing, China; 5 Nephrology Department, Beijing Hospital of Traditional Chinese Medicine, Capital Medical University, Dongcheng District, Beijing, China

**Keywords:** Acupuncture, Acute renal colic, Urinary calculi, Randomized controlled trial, Sham acupuncture

## Abstract

**Background:**

Acute renal colic caused by urinary calculi (ARCUC) has a considerable impact on the quality of life. Acupuncture might be a potential treatment option. However, the evidence is limited. We will conduct this trial to evaluate the efficacy and safety of acupuncture as adjunctive treatment to diclofenac for ARCUC.

**Methods/design:**

A total of 80 eligible patients who are diagnosed with urinary stone renal colic will be randomly allocated to the acupuncture group or the sham acupuncture group. Each patient will receive 1 session of acupuncture or sham acupuncture. The primary outcome will be the response rate of patients achieving a reduction of > 50% on visual analog score (VAS) from baseline to 10 min after treatment. Secondary outcomes will include the VAS, remedial analgesia, re-visit and admission rate, blinding assessment, credibility and expectancy, and adverse event. All patients who receive randomization will be included in the intent-to-treat analysis.

**Discussion:**

The finding of this trial will provide evidence on the efficacy and safety of acupuncture for the treatment of ARCUC. The results of this study will be published in peer-reviewed journals.

**Trial registration:**

ClinicalTrials.gov ChiCTR 1900025202. Registered on August 16, 2019.

**Supplementary Information:**

The online version contains supplementary material available at 10.1186/s13063-021-05600-2.

## Background

Acute renal colic caused by urinary calculi (ARCUC) is described as acute unbearable paroxysmal pain in the lower back or upper abdomen, with or without hematuria, nausea, and vomiting [1]. The urinary stone disease was increasingly prevalent, with a lifetime risk of about 12% in men and 6% in women [2]. The prevalence of kidney stones in China was 6.4% (6.5% in men and 5.1% in women) [3]. In the USA, more than one million patients visit the emergency department for the ARCUC every year [4]. It is described as one of the worst pains a patient could have and has a considerable impact on quality of life.

Pain relief is the primary goal in the management of patients with ARCUC [1]. NSAIDs offer effective sustained analgesia for ARCUC in the emergency department [5] and result in a lower need for rescue analgesia [6]. The 2017 update of the European Association of Urology (EAU) guidelines recommends NSAIDs as the first-line analgesic [1]. However, its clinical application is partly limited to the increased risk of major coronary events which increase with dose and duration [7, 8]. Furthermore, the onset time of NSAIDs is relatively slow, with the time to peak plasma concentration of 10–30 min after intramuscular injection [9]. More than 30% of patients did not achieve satisfactory relief of pain after had NSAIDs [5]. Adjunctive therapy with a quick analgesia effect and less adverse events is needed for patients with ARCUC.

Acupuncture is a complementary therapy from traditional Chinese medicine, which has the advantages of quick analgesia [10, 11]. A meta-analysis suggested that acupuncture may be a potential therapy for ARCUC [12]. However, to our knowledge, there is no randomized controlled trial (RCT) to measure the efficacy of acupuncture as an adjunctive treatment to NSAIDs for ARCUC. This randomized, participant-blind, sham-controlled trial is designed to evaluate the efficacy and safety of acupuncture as an adjunctive treatment to diclofenac for ARCUC.

## Methods/design

### Study design

**Fig. 1:**
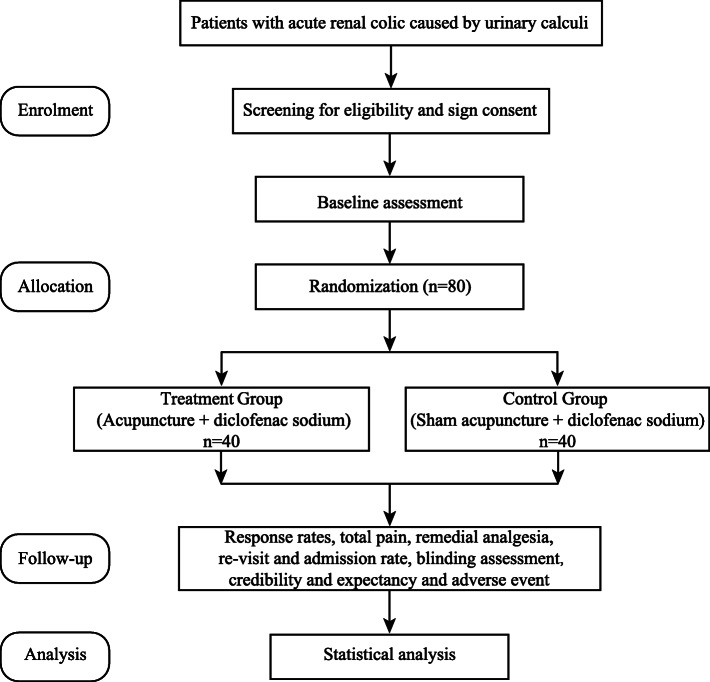
Flow diagram

This study is a single-center, randomized, participant-blind, sham-controlled trial. Each participant will receive one session of acupuncture or sham acupuncture and be followed up for 1 week after treatment. The current protocol (version v1.0, 2019.4.21) has been approved by the ethics committee of Beijing Hospital of Traditional Chinese Medicine Affiliated to Capital Medical University (ER.03.03-V1.04) and registered with the Chinese Clinical Trial Registry (ChiCTR 1900025202; registration date: 16 August 2019) before recruiting the first participant. Figure 1 shows the flow diagram of the study. The study is guided by the Declaration of Helsinki and the Standard Protocol Items: Recommendations for Interventional Trials (SPIRIT) (Additional file 1) [13].

### Patient recruitment

Participants who are diagnosed as ARCUC according to the guideline of the European Association of Urology will be recruited at Beijing Hospital of Traditional Chinese Medicine Affiliated to Capital Medical University [1]. The recruitment strategy will primarily contain advertisements on hospital social Internet media (WeChat), outpatient clinics, and the emergency room. Written informed consent will be provided by each patient through a research assistant before randomization.

### Inclusion criteria

1. Diagnosed as ARCUC according to the guideline of the European Association of Urology (2017) [1]
2. Aged 18–75 years (either sex)
3. Pain intensity of 4 or more out of 10 on a visual analog scale (VAS) [14]
4. Written informed consent

### Exclusion criteria

1. Use of any analgesia in the last 6 h
2. Allergic to diclofenac sodium, morphine, or anisodamine; history of asthma, urticaria, or allergic rhinitis ascribed to acetylsalicylic acid or other drugs containing prostaglandin synthase inhibitors
3. Congestive heart failure, acute ischemic heart disease, or peripheral vascular disease; acute cerebrovascular disease, increased intracranial pressure; renal or liver failure
4. Active digestive ulcer, pyloric obstruction, or intestinal obstruction
5. Blood system diseases such as hemophilia, coagulation disorders in patients; thrombocytopenia (< 50× 10^9^/L); using anticoagulants
6. Glaucoma, elevated intraocular pressure
7. Serious adverse reactions to acupuncture; skin infection at the acupuncture site
8. History of mental illness or substance abuse or have severe cognitive impairment
9. Pregnant or lactating

### Randomization, allocation concealment, and masking

Eighty eligible patients will be randomly assigned to the acupuncture group or the sham acupuncture group in a 1:1 ratio. The blocked randomization sequence will be computer-generated with the SAS 9.4 software by an independent professional statistician (Jing Hu, Beijing Hospital of Traditional Chinese Medicine, Capital Medical University), who is not involved in the implementation and statistical analysis of the trial. The sealed envelopes will be numbered in sequential order from 1 to 80 to hide the group assignments and be saved by a research assistant who does not take part in enrolling patients. When eligible patients are enrolled in the trial, envelopes will be successively opened by the clinical research coordinators who are responsible for enrolling the patients. Due to the responsibility of providing acupuncture and sham acupuncture, the acupuncturists will not be masked. Patients in the two acupuncture groups will be treated in a single treatment room and be blinded to which acupuncture method they would receive. In addition, outcome assessors and statisticians who perform the statistical analyses will be blinded. The group assignments will be revealed after the statistical analysis is completed.

### Interventions

Patients in both the acupuncture group and sham acupuncture group will receive 50 mg/2 mL diclofenac sodium intramuscular injection after randomization (Guangdong Bangmin Pharmaceutical Co., Ltd.). Both acupuncture and sham acupuncture will be performed by the licensed doctors of traditional Chinese medicine with at least 5 years of experience. Meanwhile, acupuncture will be performed by the licensed doctors of traditional Chinese medicine who have been trained how to locate acupoints and non-acupoints, puncture, and manipulate needles before the trial. Sterile disposable stainless steel acupuncture needles (a length of 40 mm, a diameter of 0.3 mm; Hwato, Suzhou, China) will be used. Both acupuncture and sham acupuncture treatment will only consist of 1 session treatment with 30 min. Needles will be removed if the patients suffer from any adverse events (AEs). Patients will receive 0.1 mg/kg intravenous morphine (Northeast Pharmaceutical Group Shenyang First Pharmaceutical Co., Ltd.) and 10 mg intramuscular racanisodamine (Tianjin KingYork Pharmaceutical Co., Ltd.) if they report the severity of pain more than 8 points on the VAS after puncturing the needles. No additional intravenous fluid will be administered in the first 60 min after administration of the diclofenac sodium.

### Acupuncture

**Fig. 2:**
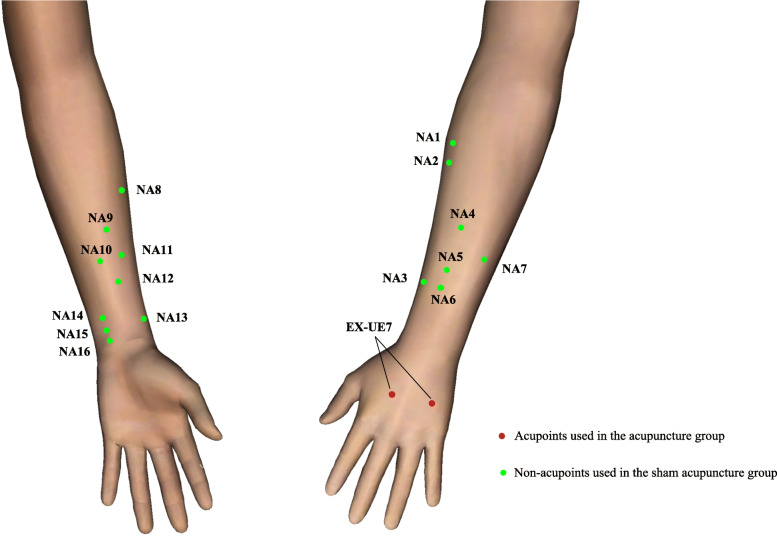
Locations of acupoints and non-acupoints. Red points are the acupoints used in the acupuncture group; green points are the non-acupoints used in the sham acupuncture group. The 16 non-acupoints will be randomly assigned to 8 subgroups. The patients in the sham acupuncture group will be assigned into 1 of these 8 subgroups, and the 2 non-acupoints in this subgroup will be used on this patient in the whole treatment period. NA, non-acupoint

Patients allocated to the acupuncture group will be punctured at the pre-specified acupoints. According to the theory of traditional Chinese medicine and clinical experience, bilateral Yaotongdian (EX-UE 7) will be used. According to the National standard of the People’s Republic of China, EX-UE 7 will contain two points on the dorsum of the hand. The one is between the second and the third metacarpal bones, and the other is between the fourth and the fifth metacarpal bones. These two points are of the same distance to the metacarpophalangeal joints and the transverse crease of the wrist. The localization of EX-UE 7 is exhibited in Fig. 2. Four needles will be used per patient, and the depth of needle insertion will be 10–15 mm. Manipulations of twirling, lifting, and thrusting will be performed on all needles for at least 30s to reach De qi (a compositional sensation including soreness, numbness, distention, and heaviness), which is believed to be an essential component for acupuncture efficacy.

### Sham acupuncture

A superficial skin penetration (1–4 mm in depth) at non-acupoints will be performed in the sham acupuncture group, without needle manipulation for De qi. Based on the search and analyses of traditional Chinese medicine reference books and acupuncture modern articles, the acupoints with effects on alleviating ARCUC or pain have been screened. After excluding these acupoints, 16 points without effects on ARCUC and pain are extracted and the locations 3 mm apart from these 16 acupoints are defined as non-acupoints, which are used in the sham acupuncture group. The locations of these non-acupoints are shown in Table 1 and Fig. 2. To make the quantity of stimulus uniform between two groups, the same number of needles for sham acupuncture will be the same as those in verum acupuncture. The 16 non-acupoints will be randomly assigned to 8 subgroups and will be recorded in predetermined computer-made randomization sealed envelopes. Each subgroup has bilateral 2 non-acupoints on the arms. The patients in the sham acupuncture group will be assigned into 1 of these 8 subgroups. This method to define non-acupoints in sham acupuncture has been used in previous acupuncture trials [15, 16].

**Table 1:** Locations of non-acupoints in the sham acupuncture group

Subgroup	Non-acupoints	Locations
1	NA 1	3 mm lateral to the Shanglian (LI9) horizontally
	NA 3	3 mm lateral to the Pianli (LI6) horizontally
2	NA 4	3 mm lateral to the Sidu (TE9) horizontally
	NA 16	3 mm lateral to the Yinxi (HT6) horizontally
3	NA 6	3 mm lateral to the Zhigou (TE6) horizontally
	NA 8	3 mm lateral to the Kongzui (LU6) horizontally
4	NA 12	3 mm lateral to the Jianshi (PC5) horizontally
	NA 2	3 mm lateral to the Xialian (LI8) horizontally
5	NA 7	3 mm lateral to the Zhizheng (SI7) horizontally
	NA 11	3 mm lateral to the Erbai (EX-UE2) horizontally
6	NA 5	3 mm lateral to the Sanyangluo (TE8) horizontally
	NA 13	3 mm lateral to the Jingqu (LU8) horizontally
7	NA 9	3 mm lateral to the Ximen (PC4) horizontally
	NA 15	3 mm lateral to the Tongli (HT5) horizontally
8	NA 14	3 mm lateral to the Lingdao (HT4) horizontally
	NA 10	3 mm internal to the Erbai (EX-UE2) horizontally

*NA* non-acupoint

### Outcomes

#### Primary outcome

The primary outcome will be the response rate after 10 min of puncturing of the needles, which is defined as the proportion of participants whose pain score on VAS reduces at least 50% compared with baseline [5].

#### Secondary outcomes

##### Total pain

The total pain will be defined by the area under the curve during the 60 min after puncturing the needles [17]. The pain will be assessed using a VAS [14] with scores ranging from 0 to 10 after 0, 1, 5, 10, 15, 20, 30, 45, and 60 min of puncturing of the needles. The bigger area under the curve indicates worse pain.

##### Response rate at other times

The proportion of participants achieving significant pain reduction will also be measured after 1, 5, 15, 20, 30, 45, and 60 min of the puncturing the needles.

##### Remedial analgesia

The number of patients who receive intravenous morphine and intramuscular racanisodamine will be recorded after 60 min of the puncturing the needles.

##### Re-visit and admission rate

The numbers of patients who re-visit the emergency department or are hospitalized will be evaluated during 72 h after puncturing the needles.

##### Blinding assessment

All patients will be asked to guess whether they receive acupuncture or sham acupuncture after acupuncture treatment to measure the patient-blinding effects.

##### Credibility and expectancy

The credibility and expectancy of patients will be assessed using the Credibility/Expectancy Questionnaire [18] after removing the needles. Items will be converted to *Z* scores before averaging, and the scale has a mean of 0.0 (SD, 1.0). The *Z* score is negative when the credibility/expectancy is below the mean and positive when it is above the mean.

##### Adverse events

All adverse events will be recorded by outcome assessors during 7 days after treatment. Based on the potential relationship between needling and adverse events, adverse events will be categorized as treatment-related or not.

**Fig. 3:**
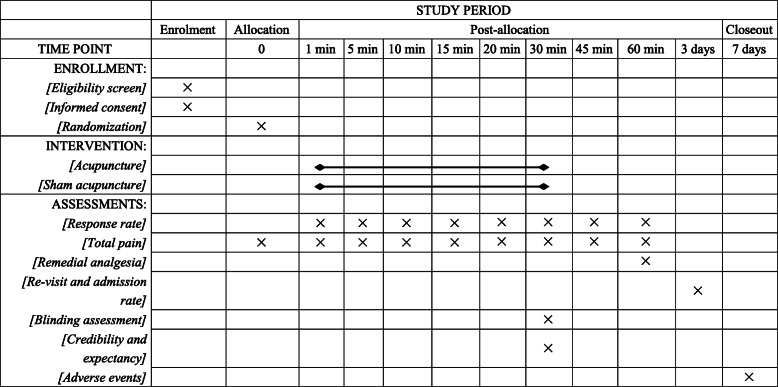
Schedule of enrollment, intervention, and assessments of this study protocol. Min, minutes

The schedule of enrolment, intervention, and assessments is shown in Fig. 3.

#### Quality control

Both paper files and electronic documents will be preserved for at least 5 years after publication. If readers have any questions, they can contact the corresponding author for access to the original data. Patient information will remain anonymous, including name, ID number, and telephone number. The protocol will be reviewed and revised by experts in acupuncture, emergency, urinary surgery, methodology, and statistics. We will perform a pre-specified standard operating procedure, which includes screening patients, improve relevant inspection, intramuscular injection of diclofenac, acupuncture, filling out the CRF, assessing outcomes, and data management. On-site monitoring will be adopted in this trial per 3 months. The ethics committee of Beijing Hospital of Traditional Chinese Medicine Affiliated to Capital Medical University will audit trial conduct per 12 months.

#### Sample size

In this study, the sample size was priori calculated. Based on the previous literature [19] and our clinical experience, the response rates in the acupuncture group and sham acupuncture group are expected to be 70% and 40%, respectively. The ratio between acupuncture group and sham acupuncture group was 1:1. A sample size of 80 patients (40 in each group) is estimated to have at least 80% power to detect difference between groups at a 2-sided significance level of 5% according to the formula: $n={\left[\frac{{\mathrm{z}}_{\alpha}\;\sqrt{4{\pi}_c\;\left(1-{\pi}_c\right)}+{\mathrm{z}}_{\beta}\;\sqrt{2{\pi}_1\;\left(1-{\pi}_1\right)+{\pi}_2\;\left(1-{\pi}_2\right)}}{\pi_1-{\pi}_2}\right]}^2$. Because there is only one session of acupuncture treatment and almost no shedding, no loss to follow-up is considered. Thus, the recruitment goal was set at 80 patients.

### Statistical analysis

Patients’ baseline characteristics will be summarized based on groups. Continuous variables will be described using the mean (standard deviation), or the median (interquartile range) if the normality assumption is violated. Student’s *t* test or Wilcoxon rank sum test (if normality is violated) will be used for comparison of continuous variables among the two groups. Categorical variables will be described using the frequency (percentage) and compared using the chi-squared test.

For the primary comparison, the chi-squared test will be used for the response rate (the proportion of participants whose pain reduced ≥ 50% compared with baseline). For the secondary outcomes, Student’s *t* test, chi-squared test, Fisher’s exact test or the Wilcoxon rank sum test will be used to test the difference of the outcomes including the total pain, remedial analgesia, re-visit and admission rate, blinding assessment, credibility and expectancy, and adverse events, between groups according to the distribution of variables. There is no interim analysis or additional analysis in this trial.

All efficacy analyses will be performed using the intention-to-treat set, which includes all randomized patients. Missing data will be dealt with the last observation carried forward (LOCF). All analyses will be performed using SPSS version 23.0 (IBM SPSS Statistics, NY, USA). The level of significance will be established at *α* < 0.05 with a two-sided test.

## Discussion

ARCUC is one of the worst pains a patient could experience and causes considerable burden for the patients and the society. This trial will evaluate the efficacy of acupuncture in improving the symptoms of ARCUC compared with sham acupuncture.

The effect of NSAIDs has been confirmed by recent meta-analysis [6]. Thus, it is recommended as first-line drugs by international guidelines [1, 20]. However, its application is partly limited for the relatively slow onset time. Previous trial carried out by Lee et al. suggested that acupuncture had a more rapid analgesic onset compared with Avafortan (3.14 ± 2.88 min versus 15.44 ± 7.55 min) [11]. Kaynar’s study also found similar phenomenon in comparing acupuncture with diclofenac [21]. Furthermore, the analgesic effect of diclofenac only last about 7 h based on its half-life elimination of 1.4 h for injection [9]. Interestingly, the persistence of the analgesic effects of acupuncture was found by an individual patient data meta-analysis [22]. Approximately 90% of the benefit of acupuncture would be sustained at 12 months. Combining acupuncture with NSAIDs may be an optional strategy for NSAIDs.

This trial meets the methodological demand for adequate randomization, allocation concealment, and blinding of patients, outcome assessors, and statisticians. Although it is difficult to set a psychologically credible yet physiologically inert control in acupuncture study, superficial insertion at non-acupoints is the most commonly used approach for administering sham treatments [23]. Moreover, all participants will be asked to guess which treatment they have received to test the patient-blinding effects. Blinding patients to interventions is more important, especially when the primary outcome is subjective, such as alleviation of pain. To avoid effects on ARCUC as far as possible, we searched points without effect on ARCUC from both ancient and modern literature. This method of selecting sham points can be seen in Alecrim [24]. There are 16 non-points, but only 2 non-points will be used to each patient in sham acupuncture group. This process of confirming non-points could further eliminate potential effects on ARCUC. After that, the prescription of acupuncture only includes one acupoint, which is very suitable for application in the emergency department due to its simplicity of operator.

This trial has limitations. First, the acupuncturists will not be masked due to the responsibility of providing the intervention. However, the patients and outcome assessors will be blinded to reduce the bias for the subjective symptom. Second, because this study is a single-center trial, the generalization of results to other medical facilities is unknown. At the end of this trial, we hope the results will provide more reliable evidence on acupuncture as an adjunctive therapy for ARCUC.

### Trial status

Protocol: version 1.0, April 21, 2019

Date opened to recruitment: March 18, 2020

Expected recruitment closure: March 31, 2021

## Supplementary Information

**Additional file 1.** Completed Standard Protocol Items: Recommendation for Interventional Trials (SPIRIT) 2013 Checklist: items addressed in this clinical trial protocol.

## Data Availability

The corresponding authors will have access to the final trial dataset. All of the individual participant data collected during the trial will be available after deidentification for anyone who wishes to access the data immediately following publication by contact the corresponding authors.

## Abbreviations

ARCUC: Acute renal colic caused by urinary calculi
AE: Adverse events
EAU: European Association of Urology
LOCF: Last observation carried forward
NSAIDs: Nonsteroidal anti-inflammatory drugs
RCT: Randomized controlled trial
SPIRIT: Standard Protocol Items: Recommendations for Interventional Trials
VAS: Visual analog scale

## References

[1] Türk C, Petřík A, Sarica K, Seitz C, Skolarikos A, Straub M, Knoll T (2016). EAU Guidelines on diagnosis and conservative management of urolithiasis. Eur Urol..

[2] Curhan GC (2007). Epidemiology of stone disease. Urol Clin North Am..

[3] Zeng G, Mai Z, Xia S, Wang Z, Zhang K, Wang L, Long Y, Ma J, Li Y, Wan SP, Wu W, Liu Y, Cui Z, Zhao Z, Qin J, Zeng T, Liu Y, Duan X, Mai X, Yang Z, Kong Z, Zhang T, Cai C, Shao Y, Yue Z, Li S, Ding J, Tang S, Ye Z (2017). Prevalence of kidney stones in China: an ultrasonography based cross-sectional study. BJU Int..

[4] Ghani KR, Roghmann F, Sammon JD, Trudeau V, Sukumar S, Rahbar H, Kumar R, Karakiewicz PI, Peabody JO, Menon M, Sun M, Trinh QD (2014). Emergency department visits in the United States for upper urinary tract stones: trends in hospitalization and charges. J Urol..

[5] Pathan SA, Mitra B, Straney LD, Afzal MS, Anjum S, Shukla D, Morley K, Hilli SAA, Rumaihi KA, Thomas SH, Cameron PA (2016). Delivering safe and effective analgesia for management of renal colic in the emergency department: a double-blind, multigroup, randomised controlled trial. Lancet..

[6] Pathan SA, Mitra B, Cameron PA (2018). A systematic review and meta-analysis comparing the efficacy of nonsteroidal anti-inflammatory drugs, opioids, and paracetamol in the treatment of acute renal colic. Eur Urol..

[7] Krum H, Swergold G, Gammaitoni A, Peloso PM, Smugar SS, Curtis SP, Brater DC, Wang H, Kaur A, Laine L, Weir MR, Cannon CP (2012). Blood pressure and cardiovascular outcomes in patients taking nonsteroidal antiinflammatory drugs. Cardiovasc Ther..

[8] Bhala N, Emberson J, Merhi A, Abramson S, Arber N, Coxib and traditional NSAID Trialists' (CNT) Collaboration (2013). Vascular and upper gastrointestinal effects of non-steroidal anti-inflammatory drugs: meta-analyses of individual participant data from randomised trials. Lancet.

[9] Dyloject (diclofenac sodium) Injection. https://www.accessdata.fda.gov/drugsatfda_docs/nda/2014/022396Orig1s000TOC.cfm. Accessed 5 August 2020.

[10] Lin LL, Wang LQ, Yang JW, Tu JF, Wang TQ, Zou X, Sun N, Liu CZ (2019). Researches status on time-effect of acupuncture. Zhongguo Zhen Jiu..

[11] Lee YH, Lee WC, Chen MT, Huang JK, Chung C, Chang LS (1992). Acupuncture in the treatment of renal colic. J Urol..

[12] Hong JH, Huang JL, Lu ZK (2017). Acupuncture therapy for calculous renal colic: a meta-analysis. Asia-Pacific Trad Med..

[13] Chan AW, Tetzlaff JM, Gøtzsche PC, Altman DG, Mann H, Berlin JA (2013). SPIRIT 2013 explanation and elaboration: guidance for protocols of clinical trials. BMJ..

[14] Hjermstad MJ, Fayers PM, Haugen DF, Caraceni A, Hanks GW, Loge JH, Fainsinger R, Aass N, Kaasa S, European Palliative Care Research Collaborative (EPCRC) (2011). Studies comparing numerical rating scales, verbal rating scales, and visual analogue scales for assessment of pain intensity in adults: a systematic literature review. J Pain Symptom Manage..

[15] Wang LP, Zhang XZ, Guo J, Liu HL, Zhang Y, Liu CZ, Yi JH, Wang LP, Zhao JP, Li SS (2011). Efficacy of acupuncture for migraine prophylaxis: a single-blinded, double-dummy, randomized controlled trial. Pain..

[16] Wang LP, Zhang XZ, Guo J, Liu HL, Zhang Y, Liu CZ (2012). Efficacy of acupuncture for acute migraine attack: a multicenter single blinded, randomized controlled trial. Pain Med.

[17] Sio TT, Le-Rademacher JG, Leenstra JL, Loprinzi CL, Rine G, Curtis A (2019). Effect of doxepin mouthwash or diphenhydramine-lidocaine-antacid mouthwash vs placebo on radiotherapy-related oral mucositis pain: the alliance A221304 randomized clinical trial. JAMA.

[18] Devilly GJ, Borkovec TD (2000). Psychometric properties of the credibility/expectancy questionnaire. J Behav Ther Exp Psychiatry..

[19] Ju BJ, Niu LL (2012). Analysis of therapeutic effect of acupuncture at Neiguan (PC 6) and Zusanli (ST 36) on acute renal colic. Zhongguo Zhen Jiu..

[20] Na YQ, Ye ZQ, Sun YH, Sun G (2014). Chinese handbook of diagnosis and treatment of urological diseases.

[21] Kaynar M, Koyuncu F, Buldu İ, Tekinarslan E, Tepeler A, Karatağ T, İstanbulluoğlu MO, Ceylan K (2015). Comparison of the efficacy of diclofenac, acupuncture, and acetaminophen in the treatment of renal colic. Am J Emerg Med..

[22] MacPherson H, Vertosick EA, Foster NE, Lewith G, Linde K, Sherman KJ, Witt CM, Vickers AJ, Acupuncture Trialists' Collaboration (2017). The persistence of the effects of acupuncture after a course of treatment: a meta-analysis of patients with chronic pain. Pain..

[23] Chen ZX, Li Y, Zhang XG, Chen S, Yang WT, Zheng XW, Zheng GQ (2017). Sham electroacupuncture methods in randomized controlled trials. Sci Rep..

[24] Alecrim-Andrade J, Maciel-Júnior JA, Carnè X, Severino Vasconcelos GM, Correa-Filho HR (2008). Acupuncture in migraine prevention: a randomized sham controlled study with 6-months posttreatment follow-up. Clin J Pain..

